# Advances in JDM: biomarker and MRI sensitivity, outcomes and steroid management in a Dutch national prospective cohort

**DOI:** 10.1093/rheumatology/keaf335

**Published:** 2025-06-18

**Authors:** Saskia R Veldkamp, Laura van der Griend, Elsbeth Noppers, Wineke Armbrust, J Merlijn van den Berg, Petra C E Hissink Muller, Esther Hoppenreijs, Sylvia Kamphuis, Judith Wienke, Femke van Wijk, Annet van Royen-Kerkhof, Marc H A Jansen

**Affiliations:** Center for Translational Immunology, University Medical Center Utrecht, Utrecht, The Netherlands; Department of Paediatric Rheumatology and Immunology, Wilhelmina Children’s Hospital, University Medical Center Utrecht, Utrecht, The Netherlands; Department of Paediatric Rheumatology and Immunology, Wilhelmina Children’s Hospital, University Medical Center Utrecht, Utrecht, The Netherlands; Department of Paediatric Rheumatology and Immunology, Beatrix Children’s Hospital, University Medical Center Groningen, Groningen, The Netherlands; Department of Paediatric Immunology, Rheumatology and Infectious Diseases, Emma Children’s Hospital, Amsterdam University Medical Centres, Location AMC, Amsterdam, The Netherlands; Department of Paediatric Rheumatology, Willem Alexander Children’s Hospital, Leiden University Medical Centre, Leiden, The Netherlands; Department of Paediatric Rheumatology, Amalia Children’s Hospital, Radboud University Medical Centre Nijmegen, Nijmegen, The Netherlands; Department of Paediatric Rheumatology, Sophia Children’s Hospital, Erasmus University Medical Centre, Rotterdam, The Netherlands; Center for Translational Immunology, University Medical Center Utrecht, Utrecht, The Netherlands; Center for Translational Immunology, University Medical Center Utrecht, Utrecht, The Netherlands; Department of Paediatric Rheumatology and Immunology, Wilhelmina Children’s Hospital, University Medical Center Utrecht, Utrecht, The Netherlands; Department of Paediatric Rheumatology and Immunology, Wilhelmina Children’s Hospital, University Medical Center Utrecht, Utrecht, The Netherlands

**Keywords:** JDM, multi-centre, biomarkers, diagnostics, outcomes, corticosteroids

## Abstract

**Objectives:**

JDM is a rare chronic inflammatory disorder of childhood. The aim of this prospective study was to describe the characteristics and outcomes of the Dutch JDM population.

**Methods:**

Demographics, clinical features, diagnostic test results and treatment were prospectively evaluated at diagnosis and during follow-up visits in JDM patients diagnosed between 2007 and 2024 in the study-coordinating centre and between 2015 and 2024 in five other tertiary referral hospitals.

**Results:**

A total of 83 patients were included (65% female). Median age at diagnosis was 6.0 years (IQR 4–9). Median follow-up was 3.2 years (IQR 1.4–5.8). The most common features at diagnosis were proximal muscle weakness (94.9%) and Gottron’s papules/sign (79.7%). While CK was abnormal in 73.1%, IFN-related biomarkers Galectin-9 and CXCL10 were elevated in 98.3% and 93.3% at diagnosis, respectively. Whole-body MRI showed muscle oedema in 30/33 (90.9%) patients. Corticosteroids could be tapered to 0.2 mg/kg at 6 months in 41.5%. Of all patients, 27 (32.5%) experienced one or more flares and 24 (28.9%) had a refractory disease course. Calcinosis occurred in 12 patients (14.5%). At last follow-up, 28.9% of patients had clinically inactive disease without medication. No patient died.

**Conclusion:**

This study demonstrates the superior diagnostic sensitivity of Galectin-9 and CXCL10 over CK, the high sensitivity of MRI and the frequent occurrence of refractory disease and flares despite treatment. Reliable predictors are needed to identify patients who can safely taper immunosuppressants versus those needing continued treatment, and who may benefit from targeted therapies initiated upfront.

Rheumatology key messages

- IFN-biomarkers Galectin-9 and CXCL10 demonstrate high diagnostic sensitivity (98% and 93%, respectively), outperforming CK (73%).
- MRI is a more reliable tool for detecting muscle inflammation at diagnosis than muscle biopsy.
- Inadequate disease control highlights the need for targeted therapies and biomarkers to guide steroid tapering.

## Introduction

JDM is a severe chronic inflammatory disease of childhood with an incidence of 2.0–4.0 per million children per year [1]. It primarily affects muscles and skin but can involve other organs too. Both the traditional diagnostic criteria by Bohan and Peter (1975) and the more recent EULAR/ACR classification criteria for idiopathic inflammatory myopathies (2017) include muscle weakness, skin manifestations, elevated muscle enzymes and, if available, muscle biopsy features [2–4]. However, recent advances such as use of MRI, myositis-specific autoantibodies (MSA) and IFN biomarkers as diagnostic tools have not yet been included in diagnostic and classification criteria, with the exception of anti-Jo-1 positivity [5–7].

With current treatment regimens, many patients experience a relapsing or chronic course. Prolonged immunosuppression, particularly corticosteroids, causes significant side effects, while persistent disease activity contributes to organ damage and complications such as calcinosis cutis [8, 9]. Evaluating patient outcomes and the role of diagnostic tools in clinical practice can help refine disease management and improve care.

This prospective study analyses JDM patient characteristics and outcomes across six Dutch academic hospitals over a total period of 16 years, providing insights into the effectiveness of biomarkers, diagnostic tools and treatment strategies.

## Methods

### Patient inclusion

Between 2015 and 2024, JDM patients were recruited at diagnosis and followed prospectively in six tertiary referral hospitals in the Netherlands, including the study-coordinating centre. Additionally, patients diagnosed at the study-coordinating centre between 2007 and 2015 were included and followed. Patients under 18 with JDM were recruited, including subtypes such as JDM sine dermatitis, amyopathic dermatomyositis (ADM), hypomyopathic dermatomyositis (HDM) and JDM with overlap features. All patients and/or parents gave written informed consent in accordance with the Declaration of Helsinki. The study was approved by the ethics committee of University Medical Center Utrecht (METC 15–191).

### Data collection

Detailed information on data collection, clinical assessments, laboratory tests and treatment is provided in Supplementary Table S1, available at *Rheumatology* online.

### Disease outcomes

Clinically inactive disease was defined as Physician’s Global Assessment (PGA) score ≤ 0.2, abbreviated Cutaneous Assessment Tool (aCAT) activity = 0, CMAS ≥ 48 (or ≥46 for children ≤4 years [10]) and creatine kinase (CK) ≤ 150 [11, 12]. All four criteria had to be met. The aCAT activity score was included due to variability in PGA use for skin symptoms. If variables were missing, disease status was determined based on the physician’s description.

Refractory disease was defined as persistent disease activity requiring treatment intensification at any time or an increase in disease activity requiring intensification within the first three months. A flare was defined as an increase in disease activity in any organ requiring treatment intensification after the first three months [13]. Treatment intensification included dose or frequency increases, medication changes or new systemic therapies (excluding topical treatments).

### Statistical analysis

Statistical analyses were performed using IBM SPSS Statistics, version 29.0.1 (IBM Corp., Armonk, N.Y., USA), with details provided in Supplementary Data S1, available at *Rheumatology* online.

## Results

A total of 83 patients were included in this study (flow diagram in Supplementary Fig. S1, available at *Rheumatology* online). Based on the total of 82 newly diagnosed patients between June 2015 and October 2023 and a median population of 3.36 million children per year in the Netherlands in 2015–2024 [14], the incidence was established at 2.9 cases/million/year.

Demographic data are summarized in Table 1. Median duration between onset of symptoms and diagnosis was 3.0 months (IQR 1.3–5.9 months, range 7 days–3.8 years). Patients were followed for a median of 3.2 years (IQR 1.4–5.8) with a median number of visits of 11 (IQR 6–16). Family history of autoimmune disease was present in 36/83 patients (43.4%) (Supplementary Table S2, available at *Rheumatology* online).

**Table 1.** Patient characteristics at diagnosis and 6 months

Demographics	*N* = 83
Female, *N* (%)	54 (65.1)
Male, *N* (%)	29 (34.9)
Age (years) at diagnosis, median (IQR, range)	6.0 (4–9, 1–17)
Ethnicity, *N* (%)	
West-European	57 (68.7)
Other European	2 (2.4)
Turkish	6 (7.2)
Other European	18 (21.7)
JDM subtype, *N* (%)	
Classic JDM	74 (89.2)
JDM sine dermatitis	5 (6.0)
Hypomyopathic DM (HDM)	2 (2.4)
Amyopathic DM (ADM)	2 (2.4)

Clinical features, *N* (%)		Diagnosis *N* = 79	6-month follow-up *N* = 69
Muscle	Proximal muscle weakness	75 (94.9)	18 (26.1)
	Myalgia	51 (64.6)	5 (7.2)
	Non-proximal muscle weakness	34 (43.0)	3 (4.3)
	Dysphagia due to myositis	18 (22.8)	1 (1.4)
	Dysphonia due to myositis	16 (20.3)	1 (1.4)
	Joint contractures	11 (13.9)	5 (7.2)
	Muscle atrophy	10 (12.7)	3 (4.3)
	Reduced cough strength	2 (2.5)	0 (0.0)
	Eye movement disorder	1 (1.3)	0 (0.0)
	Fasciitis	1 (1.3)	0 (0.0)
Skin^a^	Gottron's papules/sign	63 (79.7)	26 (39.4)
	Malar or facial erythema	60 (75.9)	17 (25.8)
	Heliotrope rash	54 (68.4)	19 (28.8)
	Periungual capillary loop changes^b^	40 (50.6)	10 (15.2)
	Subcutaneous oedema	31 (39.2)	3 (4.5)
	Other non-specified erythema^c^	19 (24.1)	4 (6.1)
	Livedo reticularis	17 (21.5)	1 (1.5)
	Non-sun exposed erythema	16 (20.3)	4 (6.2)
	‘Shawl’ sign rash	12 (15.2)	2 (3.0)
	Linear extensor erythema	11 (13.9)	3 (4.5)
	‘V’ sign rash	7 (8.9)	2 (3.0)
	Ulceration	7 (8.9)	2 (3.0)
	Other skin symptoms^d^	5 (6.3)	1 (1.5)
	Other vasculopathic lesions^e^	5 (6.3)	5 (7.8)
	Erythroderma	4 (5.1)	0 (0.0)
	Poikiloderma vascular atrophicans	3 (3.8)	0 (0.0)
	Mechanic's hands	3 (3.8)	1 (1.5)
	Cuticular overgrowth	3 (3.8)	1 (1.5)
	Alopecia	3 (3.8)	0 (0.0)
	Mucous membrane lesions	2 (2.5)	0 (0.0)
	Calcinosis	1 (1.3)	2 (3.0)
	Panniculitis	1 (1.3)	0 (0.0)
	Lipoatrophy	1 (1.3)	0 (0.0)
	Pityriasis rubra pilaris^f^	1 (1.3)	0 (0.0)
	Scleroderma-like skin lesions	1 (1.3)	0 (0.0)
Skeletal	Arthritis	9 (11.4)	1 (1.4)
	Arthralgia	4 (5.1)	1 (1.4)
	Tendinitis	2 (2.5)	0 (0.0)
Respiratory	Active interstitial lung disease (ILD)	3 (3.8)	2 (2.9)
	Respiratory muscle weakness (dyspnea) without ILD	2 (2.5)	0 (0.0)
	Impaired lung function due to myositis or (respiratory) muscle damage	1 (1.3)	0 (0.0)
Gastrointestinal	Abdominal pain	6 (7.6)	1 (1.4)
	Globus	1 (1.3)	0 (0.0)
	Vomiting	1 (1.3)	0 (0.0)
	Gastrointestinal dysmotility, constipation, diarrhea	0 (0.0)	1 (1.4)
Cardiovascular	Raynaud's phenomenon	8 (10.1)	1 (1.4)
	Myocarditis	1 (1.3)	0 (0.0)
	Nail clubbing	1 (1.3)	0 (0.0)
Other	Fatigue	55 (69.6)	16 (23.2)
	Irritability	25 (31.6)	2 (2.9)
	Fever >38.0°C	11 (13.9)	1 (1.4)
	Unintentional weight loss >5%	11 (13.9)	0 (0.0)
	Headache	3 (3.8)	3 (4.3)
	Lymphadenopathy	2 (2.5)	0 (0.0)
	Dysarthria	1 (1.3)	0 (0.0)

a *N* = 66 for data on skin features at 6-month follow-up.

b Nailfold capillaroscopy was not consistently carried out; the determination of periungual capillary loop changes relied on the observation of periungual erythema as well.

c Other non-specified erythema included other unspecified erythema, erythematous patch, maculopapular exanthema, erythematous lesions and photosensitivity.

d Other skin symptoms included vesicles, depigmentation, hyperkeratosis and subcutaneous nodules.

e Other vasculopathic lesions included vasculopathic lesions, petechiae and telangiectasias.

f This patient was diagnosed as Wong-type dermatomyositis [15].

### Clinical characteristics

Data at diagnosis were available for 79 patients and at 6-month follow-up (median 6.5 months, IQR 5.6–7.1) for 69 patients; missing data were due to lack of clinical visits, incomplete assessments or diagnosis abroad. Clinical characteristics are reported at diagnosis and 6-month follow-up (Table 1).

#### Muscle

The most common muscle features at diagnosis were proximal muscle weakness (94.9%) and myalgia (64.6%). Of the four patients without muscle weakness at diagnosis, two showed biochemical (CK of 972 and 3746 U/l) and radiological (MRI) evidence of myositis and were classified as HDM. The other two, with typical skin manifestations, were classified as ADM. The median CMAS score at diagnosis was 31 (IQR 19–42, range 1–52; *n* = 64) and improved within 6 months to 45 (IQR 42–47; *n* = 45).

#### Skin

The most frequent skin features at diagnosis were Gottron’s papules/sign (79.7%), malar or facial erythema (75.9%) and heliotrope rash (68.4%). At diagnosis, six patients did not have the pathognomonic heliotrope rash or Gottron’s papules/sign, although one developed both manifestations during a flare eight years later. The median aCAT activity score at diagnosis was 4 (IQR 3–5) and improved to 1 (IQR 0–2) at 6-month follow-up. At both timepoints, the median aCAT damage score was 0 (range at diagnosis 0–5, range at 6 months 0–1).

#### Extramuscular/-cutaneous manifestations

The most frequent extramuscular/-cutaneous manifestations at diagnosis were fatigue (69.6%), irritability (31.6%), fever (13.9%), weight loss (13.9%) and arthritis (11.4%). In three patients, interstitial lung disease (ILD) was detected. All three were diagnosed with JDM with antisynthetase syndrome or overlap features based on clinical features and autoantibodies.

### Diagnostic investigations

**Figure 1.**
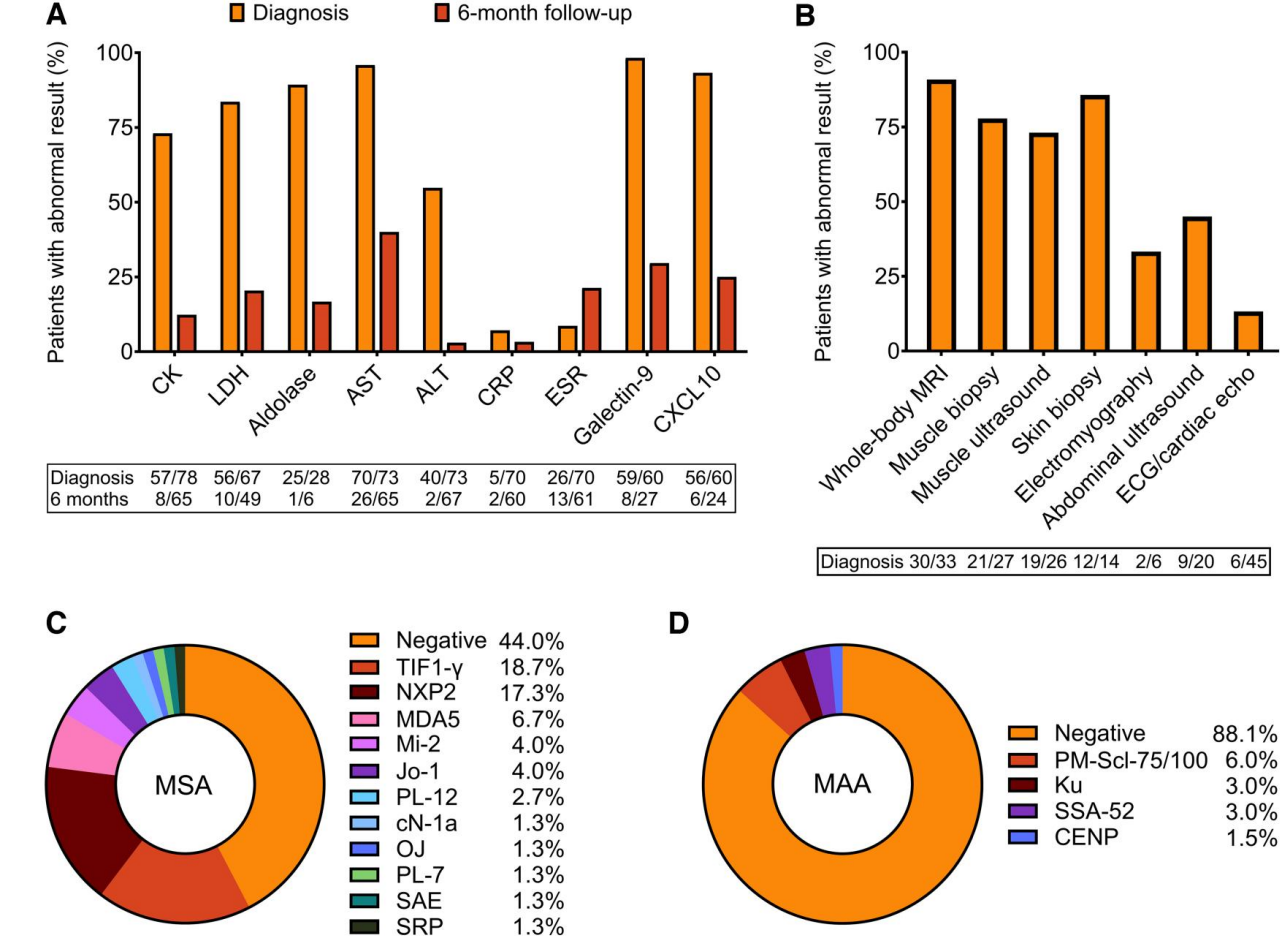
Diagnostic investigations and autoantibody profiles. (**A**) Percentages of patients with abnormal blood test results at diagnosis and 6-month follow-up. Numbers below the figure indicate the number of patients with abnormal results out of the total number of patients tested for each test (abnormal/total). (**B**) Percentages of patients with abnormal findings at diagnosis on imaging, histology, electromyography and electro/-echocardiography. Numbers below the figure indicate the number of patients with abnormal results out of the total number of patients investigated for each investigation (abnormal/total). (**C**) Frequencies of myositis-specific autoantibodies (MSA), tested in 75 patients. Percentages add up to 102.5% due to two patients testing positive for >1 MSA. (**D**) Frequencies of myositis-associated autoantibodies (MAA), tested in 67 patients. Percentages add up to 101.5% due to one patient testing positive for >1 MAA

Percentages of abnormal diagnostic investigations are shown in Fig. 1A and B, with raw blood test values at diagnosis shown in Supplementary Fig. S2, available at *Rheumatology* online.

CK was most frequently tested and was abnormal in 73.1% at diagnosis (range 44–26 437 U/l). Patients with normal CK had significantly better CMAS scores (median 38 vs 25, *P* = 0.004). Among those with normal CK but with a CMAS of ≤39, indicating mild-to-moderate impairment [16], the median time from symptom onset to diagnosis was longer (6.3 vs 2.0 months, *P* = 0.028), suggesting CK normalization in some due to muscle degradation. Six months post-diagnosis, CK had normalized in 87.7% of patients.

Galectin-9 and CXCL10 were elevated at diagnosis in 98.3% and 93.3% of patients, respectively. At 6-month follow-up, Galectin-9 and CXCL10 had normalized in 70.4% and 75.0% of patients with available measurements, respectively. The only patient with normal Galectin-9 and CXCL10 levels at diagnosis had minimal clinical and biochemical evidence of muscle weakness (CK 146 U/l, CMAS 48), a normal muscle ultrasound, MRI and biopsy, and predominant skin involvement (aCAT activity score 6).

Whole-body MRI showed muscle oedema consistent with myositis in 30/33 (90.9%) patients. Of the remaining three patients, one is described above, one had a CK of 308 U/l, CMAS of 45 and a negative biopsy, and one had strong skin involvement, a CK of 211 U/l and CMAS of 35; no muscle biopsy was performed.

Twenty-eight muscle biopsies were performed in 27 patients (*n* = 15 needle biopsy, *n* = 4 open biopsy, *n* = 9 unknown), before treatment initiation. In one patient, no tissue was obtained, leaving 27 biopsies with evaluable tissue. Of these, 21 (77.8%) were abnormal (suggestive of inflammatory myopathy). Of the six patients with a normal muscle biopsy, two have been described above, three had clear clinical, biochemical and radiological evidence of myositis as well as typical skin changes, and one had insufficient clinical data available.

Abnormalities detected by abdominal ultrasound were predominantly hepatomegaly. Observed cardiac abnormalities included right bundle branch block morphology, dilated vena cava inferior, respiratory arrhythmia, left ventricle hypertrophy and myocarditis. In all cases, no additional treatment was considered necessary.

#### Autoantibodies

Antinuclear antibodies (ANAs) were positive in 38/80 patients (47.5%), MSA in 56.0% (42/75) and myositis-associated autoantibodies (MAA) in 11.9% (8/67). Individual MSA and MAA frequencies are shown in Fig. 1C and D.

The prevalence of the most common MSA in our cohort in relation to commonly reported associated clinical features are summarized in Supplementary Table S3, available at *Rheumatology* online. Anti-TIF1-γ+ patients often presented with severe cutaneous disease, including skin ulcerations, while anti-NXP2+ patients frequently had severe muscle disease, as well as calcinosis. Several patients exhibited overlap features associated with their autoantibody profile (Supplementary Table S4, available at *Rheumatology* online).

### Treatment

Patients were treated following the SHARE guidelines, which recommend high-dose steroids in combination with MTX, and if necessary intensification with other immunomodulatory or -suppressive medications (Supplementary Table S5, available at *Rheumatology* online) [5]. All three patients with ILD received additional treatment: one with mycophenolate mofetil and rituximab, another with rituximab and the third with rituximab and IVIGs. Two patients underwent an autologous haematopoietic stem cell transplantation due to severe refractory disease, which resulted in drug-free remission in one of them. The median duration to complete medication discontinuation was 3.4 years (IQR: 2.2–4.1 years, range 5.5 months–12.7 years).

#### Steroid tapering

The majority (78.5%) initially received high-dose intravenous steroid pulse therapy, followed by oral steroids at 1–2 mg/kg with a maximum dose of 60 mg. Tapering was guided by the PRINTO protocol and the physician’s clinical judgement [17]. The median duration to steroid discontinuation was 12.9 months (IQR 8.2–17.6, range 1.4–41.7). At 6-month follow-up, the median dose was 0.23 mg/kg (IQR 0.12–0.40, range 0.0–0.85). Supplementary Fig. S3, available at *Rheumatology* online, depicts the distribution of oral steroid doses at this timepoint.

A subgroup of 10 patients tapered off oral steroids completely within 6 months. This group included seven with classical JDM, and one each with ADM, HDM and JDM sine dermatitis. They showed no significant differences in PGA, CMAS or aCAT activity scores at diagnosis or 6 months (Supplementary Table S6, available at *Rheumatology* online). CK was higher at 6 months but within normal range (*P* = 0.008). One patient experienced a (skin) flare and was started on hydroxychloroquine. At the last follow-up (median 15.0 months, range 5.5–41.1), nine had clinically inactive disease, while one patient (follow-up 7.3 months) had active disease (PGA 2).

#### Toxicity

Frequencies of reported side effects are shown in Supplementary Table S7, available at *Rheumatology* online. Decreased bone density led to a femur fracture in one patient and to osteonecrosis in another. The recorded anaphylactic or allergic reactions were all due to IVIG transfusions. Treatment-related cytopenias included lymphopenia, pancytopenia and macrocytic anaemia due to MTX and/or cyclophosphamide.

### Disease course

#### Refractory disease

Of all patients, 24 (28.9%) required treatment intensification due to refractory disease, with 21 cases starting this intensification within the first 3 months post-diagnosis. The types of tissue involved are shown in Supplementary Fig. S4A, available at *Rheumatology* online. Nine patients also had one or more flares during follow-up.

#### Flares

Of all patients, 27 (32.5%) experienced one or more flares during follow-up. Eighteen patients experienced a single flare, six patients experienced two flares, two patients experienced four flares and one patient experienced five flares. The types of tissue involved are shown in Supplementary Fig. S4B, available at *Rheumatology* online. The first flare occurred with a median time of 23.2 months (IQR 10.3–44.7, range 3.2–142.3) after diagnosis.

Of all flares, 81.4% (35/43) occurred while the patient was still on any type of medication. Seventeen flares (39.5%) happened during or within 6 months after tapering of medication (i.e. dose or frequency reduction), with 12 of these occurring during oral steroid tapering (all ≤0.5 mg/kg). Sixteen flares (37.2%) occurred within 6 months after discontinuation of a type of medication, including oral steroids in eight cases.

#### Calcinosis

Calcinosis was present at diagnosis in one patient and developed during the disease course in 11 others (12/83, 14.5%). The median time to onset was 1.3 years (IQR 8.0 months–3.1 years, range 0–5.3 years) post-diagnosis. Evolution and treatment details per patient are shown in Supplementary Table S8, available at *Rheumatology* online.

#### Disease status at last follow-up

Overall, at last follow-up, 43/83 patients (51.8%) had clinically inactive disease according to the predefined criteria, 24 (28.9%) of whom were off medication. An additional 24 patients (28.9%) did not fully meet these criteria but were considered to be clinically inactive by the treating physician. This discrepancy was primarily due to marginally elevated CK levels (150–200 U/l), PGA scores of 0.5 and missing CMAS scores.

Out of the 67 patients who were considered to be clinically inactive by the physician, 18 (26.9%) patients had complaints that were considered to be due to damage. These included nine patients with cutaneous damage, such as atrophic lesions, dyspigmentation and poikiloderma vasculare atrophicans, seven patients with proximal muscle weakness, four with myalgia and two with muscle atrophy. No deaths occurred.

## Discussion

This prospective cohort study provides the first comprehensive report on the Dutch JDM population, showing similarities to other cohorts in incidence, female/male ratio, age at diagnosis and time to diagnosis [18–20]. Our findings contribute to the global understanding of this rare and heterogeneous disease and offer key insights into current diagnostic and treatment practices.

First, Galectin-9 and CXCL10 were elevated at diagnosis in 98.3% and 93.3% of patients, respectively. These results indicate that these IFN-induced serum proteins, previously validated as robust biomarkers of disease activity and routinely used in the Netherlands for disease monitoring, also have high diagnostic sensitivity [21]. In contrast, CK, which is included in the diagnostic criteria, was elevated in only 73.1% of cases, consistent with prior studies [19, 20]. Normal CK levels were linked to milder disease but also delayed diagnosis. As IFN-induced proteins, Galectin-9 and CXCL10 likely reflect ongoing inflammation more accurately than CK, a product of muscle injury. However, their specificity is limited, as they are also elevated in other IFN-driven diseases and (viral) infections [22–24]. In our previous study, both biomarkers were low in healthy controls and non-inflammatory neuromuscular disease, but elevated in SLE and adult myositis, suggesting high sensitivity for detecting IFN-driven inflammation but limited disease specificity for JDM [21]. Future studies are warranted to uncover their distinct roles as biomarkers.

Second, whole-body MRI demonstrated high sensitivity for detecting muscle inflammation at diagnosis (90.9%), outperforming muscle biopsy (77.8%), likely due to sampling errors. These findings align with a multi-centre study of 384 patients [25]. MRI-guided biopsy may reduce false-negative biopsy rates [26].

Third, despite early aggressive treatment per SHARE guidelines, half of the patients required treatment intensification for refractory disease or flares. These outcomes indicate inadequate disease control and highlight the need for more effective, targeted therapies. Significant toxicity was reported, emphasizing the importance of limited and tapered steroid use. Although our cohort had a shorter median steroid duration (12.9 months) than the proposed tapering protocol by PRINTO (24 months), only 41.5% reached the recommended 0.2 mg/kg dose by 6 months [17]. Notably, 10 patients discontinued steroids within 6 months without increased flares, yet nearly half of flares (20/43) occurred closely after steroid dose reduction or discontinuation, suggesting subclinical disease in some. Reliable biomarkers are needed to detect this subclinical inflammation and guide safer tapering and discontinuation.

Strengths of this study include its prospective multi-centre design, standardized biomarker collection and inclusion of rarer JDM subtypes, reflecting the full clinical spectrum of the disease. By reporting clinical features and medication use at 6 months follow-up, we provide a relevant benchmark for treat-to-target strategies and clinical trial design. Limitations include a limited median follow-up time, and small sample sizes for MSA subtypes, restricting subgroup analysis. Additionally, variations in assays over time and across centres may have led to false-negative MSA results. Finally, inconsistent documentation of outcomes like toxicity, damage and abnormal nailfold capillaries likely led to underestimation of these factors in our cohort.

In conclusion, this first prospective study of the Dutch JDM population supports the use of IFN-induced biomarkers Galectin-9 and CXCL10 and whole-body MRI in JDM diagnostics. The high frequency of refractory disease, flares and steroid-related toxicity underscores the necessity for a more personalized treatment approach. This approach should incorporate biomarkers that can predict treatment response, guide steroid tapering and identify candidates for targeted therapies.

## Supplementary Material

keaf335_Supplementary_Data

## Data Availability

All data of this study are available to the scientific community upon reasonable request.
