# Supplementary material for: Advances in JDM: biomarker and MRI sensitivity, outcomes and steroid management in a Dutch national prospective cohort
Source: Rheumatology (Oxford). 2025 Jun 18;64(11):5622–8. doi: 10.1093/rheumatology/keaf335 (PMC12596064; doi:10.1093/rheumatology/keaf335)
Supplement: keaf335_Supplementary_Data [file keaf335_supplementary_data.docx]

**Supplementary Data S1**

- Supplementary methods
  - Supplementary Data S1. Statistical analysis
- Supplementary Tables
  - Supplementary Table S1. Data collection
  - Supplementary Table S2. Autoimmune disease in family history
  - Supplementary Table S3. Clinical associations of common Myositis-Specific Autoantibodies
  - Supplementary Table S4. Autoantibodies and overlap features
  - Supplementary Table S5. Medication use
  - Supplementary Table S6. Comparison of clinical characteristics in patients who were able vs. unable to discontinue oral steroids within 6 months
  - Supplementary Table S7. Toxicity
  - Supplementary Table S8. Calcinosis onset, duration and treatment
- Supplementary Figures
  - Supplementary Figure S1. Flow diagram of study inclusions
  - Supplementary Figure S2. Blood test results at diagnosis
  - Supplementary Figure S3. Oral steroid dose at 6-month follow-up
  - Supplementary Figure S4. Refractory disease and flares
- References

**Supplementary methods**

**Supplementary Data S1. Statistical analysis**

Outcomes were reported as absolute and percentage frequencies or as medians with interquartile ranges (IQR, 25^th^–75^th^ percentiles). Continuous variables were compared between groups using independent samples t-tests or Mann-Whitney U Tests. Categorical variables were compared using Chi-square tests. Statistical significance was set at p < 0.05. Analyses were conducted using SPSS Statistics (Version 29.0.1).

**Supplementary Tables**

| **Supplementary Table S1. Data collection** | | | | |  |
| --- | --- | --- | --- | --- | --- |
| **Type of data** | | **Details** | | | **Timepoints** |
| **Demographics** | | Gender, ethnicity (parent-reported), age, symptom onset, family history, medical history | | | Diagnosis |
| **Clinical assessments** | | Childhood Myositis Assessment Scale (CMAS, 0-52, max score representing normal muscle strength and function) [1];  Abbreviated Cutaneous Assessment Tool (aCAT, activity: 0-17, damage: 0-11) [2];  Physician’s Global Assessment (PGA) of disease activity (0-10) | | | Diagnosis and follow-up |
| **Blood tests** | | Creatine kinase (CK), ULN 145 (girls) or 170 (boys) U/L;  Lactate dehydrogenase (LDH), ULN 235-935 U/L, depending on age;  Aldolase ULN 7.6 U/L;  Aspartate transaminase (AST), ULN 30 (girls) or 35 (boys) U/L;  Alanine transaminase (ALT), ULN 35 (girls) or 45 (boys) U/L;  CRP, ULN 10 mg/L;  ESR, ULN 15 (girls) or 20 (boys) mm/h;  Galectin-9 (ULN 5073 pg/mL) and CXCL10 (ULN 805 pg/mL) measured in routine diagnostics (n=24, n=20 at diagnosis; n=27, n=24 at 6 months) and in research (n=41 at diagnosis) [3] | | | Diagnosis and follow-up |
| **Additional investigations** | | Whole-body MRI, muscle biopsy, muscle ultrasound, skin biopsy, electromyography, abdominal ultrasound, electrocardiography and echocardiography | | | Diagnosis |
| **Treatment** | | Medication use, toxicity | | | Diagnosis and follow-up |
| ULN = upper limit of normal; MRI = Magnetic Resonance Imaging | | | | | |
| **Supplementary Table S2. Autoimmune disease in family history** | | |  |  |  |
| **Autoimmune disease** | | **No. of patients (%)** |  |  |  |
| Rheumatoid Arthritis | | 12 (14.5) |  |  |  |
| Thyroid-related^a^ | | 12 (14.5) |  |  |  |
| Psoriasis | | 7 (8.4) |  |  |  |
| Inflammatory Bowel Disease | | 5 (6.0) |  |  |  |
| Diabetes Mellitus | | 5 (6.0) |  |  |  |
| Sarcoidosis | | 4 (4.8) |  |  |  |
| Multiple Sclerose | | 4 (4.8) |  |  |  |
| Juvenile Idiopathic Arthritis | | 4 (4.8) |  |  |  |
| Dermatomyositis | | 2 (2.4) |  |  |  |
| Ankylosing spondylitis (AS) | | 2 (2.4) |  |  |  |
| Vitiligo | | 2 (2.4) |  |  |  |
| Guillain–Barré syndrome | | 1 (1.2) |  |  |  |
| Stevens Johnsons Syndrome | | 1 (1.2) |  |  |  |
| Lichen Planus | | 1 (1.2) |  |  |  |
| Polymyalgia Rheumatica | | 1 (1.2) |  |  |  |
| Parkinson’s Disease | | 1 (1.2) |  |  |  |
| Arthritis psoriatica | | 1 (1.2) |  |  |  |
| Scleroderma | | 1 (1.2) |  |  |  |
| Systemic Lupus Erythematosus | | 1 (1.2) |  |  |  |
| Sjögren’s Syndrome | | 1 (1.2) |  |  |  |

N=83. ^a^ Thyroid-related diseases were not further specified

| **Supplementary Table S3. Clinical associations of common Myositis-Specific Autoantibodies** | | | | | |  |
| --- | --- | --- | --- | --- | --- | --- |
|  | **anti-TIF1γ+**  **(n=14)** | **anti-NXP2+ (n=12^a^)** | **anti-MDA5+ (n=5)** | **anti-Mi-2+**  **(n=3)** | **MSA-negative (n=33)** |  |
| Severe muscle weakness^b^ | 2 (14%) | 7 (58%) | 1 (20%) | 2 (67%) | 14 (42%) |  |
| Subcutaneous edema | | 8 (57%) | 7 (58%) | 2 (40%) | - | 14 (42%) |
| Dysphagia and/or dysphonia | | 4 (29%) | 5 (42%) | - | 1 (33%) | 14 (42%) |
| Joint contractures | 2 (14%) | 4 (33%) | 1 (20%) | - | 9 (27%) |  |
| Severe cutaneous disease^c^ | 7 (50%) | 3 (25%) | - | 1 (33%) | 8 (24%) |  |
| Skin ulceration | 4 (29%) | 2 (17%) | - | - | 1 (3%) |  |
| Calcinosis | | - | 3 (25%) | 1 (20%) | - | 6 (18%) |
| Interstitial lung disease | - | - | - | - | 1 (3%) |  |
| ADM | - | - | 1 (20%) | - | 1 (3%) |  |

^a^ N=13, but one patient did not have sufficient clinical data. ^b^ Severe muscle weakness was defined as a CMAS score ≤ 30 [1]. ^c^ Severe cutaneous disease was defined as an aCAT activity score ≥ 6. MSA = myositis-specific autoantibody, ADM = amyopathic dermatomyositis. We did not perform statistical analyses due to small sample sizes.

| **Supplementary Table S4. Autoantibodies and overlap features** | | |
| --- | --- | --- |
| **Patient** | **MSA or MAA** | **Overlap features** |
| 1 | Anti-PL-7, anti-SSA-52 | Interstitial lung disease, Raynaud’s phenomenon, esophagus dysmotility |
| 2 | Anti-Jo-1, anti-PM-Scl-75/100 | Generalized pityriasis rubra pilaris (Wong-type JDM), mechanic’s hands, Raynaud’s phenomenon |
| 3 | Anti-Jo-1, anti-SSA-52, anti-CENP-B | Interstitial lung disease, arthritis, mechanic’s hands |
| 4 | Anti-PM-Scl-75/100 | Arthritis, mechanic’s hands, Raynaud’s phenomenon, sclerotic skin, calcinosis at onset |
| 5 | Anti-PM-Scl-75/100 | Interstitial lung disease, arthritis, myocarditis, nail clubbing |

MSA = myositis-specific autoantibody, MAA = myositis-associated autoantibody

| **Supplementary Table S5. Medication use** | | | | |
| --- | --- | --- | --- | --- |
|  | **Diagnosis**  N=79  *N (%)* | **6-month follow-up**  N=69  *N (%)* | | **During disease course**  N=83  *N (%)* |
| Oral steroids | 74 (93.7) | 55 (79.7) | | 77 (92.8)^a^ |
| Methotrexate | 69 (87.3) | 58 (84.1) | | 76 (91.6) |
| High-dose intravenous steroid pulse therapy | 62 (78.5) | 0 (0.0) | | 65 (78.3) |
| IVIG | 12 (15.2)^b^ | 13 (18.8) | | 37 (44.6) |
| Hydroxychloroquine | 6 (7.6) | 7 (10.1) | | 14 (16.9) |
| Mycophenolate mofetil | 2 (2.5) | 5 (7.2) | | 17 (20.5) |
| Cyclophosphamide | 0 (0.0) | 1 (1.4) | | 10 (12.0) |
| Infliximab | 0 (0.0) | 1 (1.4) | | 4 (4.8) |
| Rituximab | 0 (0.0) | 0 (0.0) | 7 (8.4) | |
| Adalimumab | 0 (0.0) | 0 (0.0) | 1 (1.2) | |
| Tacrolimus | 0 (0.0) | 0 (0.0) | 6 (7.2) | |
| Tofacitinib | 0 (0.0) | 0 (0.0) | 1 (1.2) | |
| Autologous HSCT | 0 (0.0) | 0 (0.0) | 2 (2.4) | |
| ^a^ Differences in available data at diagnosis (n=79) and during disease course (n=83) explain the lower percentage during disease course, despite a higher absolute number. ^b^ IVIG was started as standard initial treatment in one of the participating centers. Start of systemic medication was deemed unnecessary at diagnosis for two patients; one with ADM and one with JDM sine dermatitis who was started on IVIG four months post-diagnosis. HSCT = hematopoietic stem cell transplantation. | | | | |

| **Supplementary Table S6. Comparison of clinical characteristics in patients who were able vs. unable to discontinue oral steroids within 6 months** | | | |
| --- | --- | --- | --- |
|  | **Patients off oral steroids at 6 months**  *median (IQR)* | **Patients on oral steroids at 6 months**  *median (IQR)* | *p-value* |
| **At diagnosis^a^** |  |  |  |
| PGA score | 5 (2.4 – 6) | 6 (4.5 – 7.0) | 0.107 |
| CMAS score | 35 (25 – 45) | 30 (17 – 39) | 0.333 |
| aCAT activity score | 4 (3.3 – 4) | 4 (3 – 6) | 0.349 |
| CK | 1041 (233 – 2382) | 1167 (160 – 4948) | 0.733 |
| **6-month follow-up^b^** |  |  |  |
| PGA score | 0.5 (0 – 1) | 1 (0 – 2) | 0.232 |
| CMAS score | 45 (43 – 48) | 45 (41 – 47) | 0.647 |
| aCAT activity score | 0.5 (0 – 1) | 1 (0 – 2) | 0.471 |
| CK | 109 (103 – 118) | 77 (62 – 117) | **0.008** |

^a^ N=10 (off steroid group) and n=60 (on steroid group). ^b^ N=10 (off steroid group) and n=55 (on steroid group). PGA = Physician’s Global Assessment, aCAT = abbreviated Cutaneous Assessment Tool, CMAS = Childhood Myositis Assessment Scale, CK = creatine kinase (U/L).

| **Supplementary Table S7. Toxicity** | |
| --- | --- |
| **Toxicity** | **No. of patients (%)** |
| Cushing syndrome | 39 (47.6) |
| Nausea | 32 (39.0) |
| Headache | 16 (19.5) |
| Hypertension | 11 (13.4) |
| Mood swings | 12 (13.4) |
| Fever | 9 (11.0) |
| Issues related to bone density | 8 (9.8) |
| Abdominal pain | 7 (8.5) |
| Increased levels of AST/ALT | 7 (8.5) |
| Vomiting | 6 (7.3) |
| Anaphylactic/allergic reaction | 5 (6.1) |
| Alopecia | 4 (4.9) |
| Varicella Zoster reactivation | 4 (4.9) |
| Cytopenia | 4 (4.9) |
| Diabetes mellitus | 2 (2.4) |

N=82, as one patient did not receive any treatment.

| **Supplementary Table S8. Calcinosis onset, duration and treatment** | | | | |
| --- | --- | --- | --- | --- |
| **Patient** | **Onset calcinosis (from diagnosis)** | **Duration calcinosis** | **Evolution** | **Treatment started specifically for calcinosis^a^** |
| 1 | 0.2 years | Reported once | Resolved | None |
| 2 | 0.5 years | 1.7 years | Resolved | None |
| 3 | 0.0 years | 0.5 years | Resolved | IVIG |
| 4 | 1.3 years | 3.3 years | Progressive | Shockwave therapy and pamidronate i.v. |
| 5 | 1.3 years | 1.5 years | Unknown | Adalimumab |
| 6 | 1.1 years | 2.9 years | Resolved | None |
| 7 | 0.8 years | 1.7 years | Progressive, then resolved | High dose i.v. steroid pulse therapy and switch MTX to MMF |
| 8 | 5.3 years | 3.3 years | Progressive, then regressive | None |
| 9 | 2.4 years | 3.7 years | Resolved | None |
| 10 | 2.0 years | 4.5 years | Progressive, then stable | Pamidronate i.v. |
| 11 | 1.3 years | 2.7 years | Stable | None |
| 12 | 4.4 years | Reported once | Resolved | None |
| ^a^ Excludes immunosuppressive treatment given for overall disease management including refractory disease. | | | | |

**Supplementary Figures**


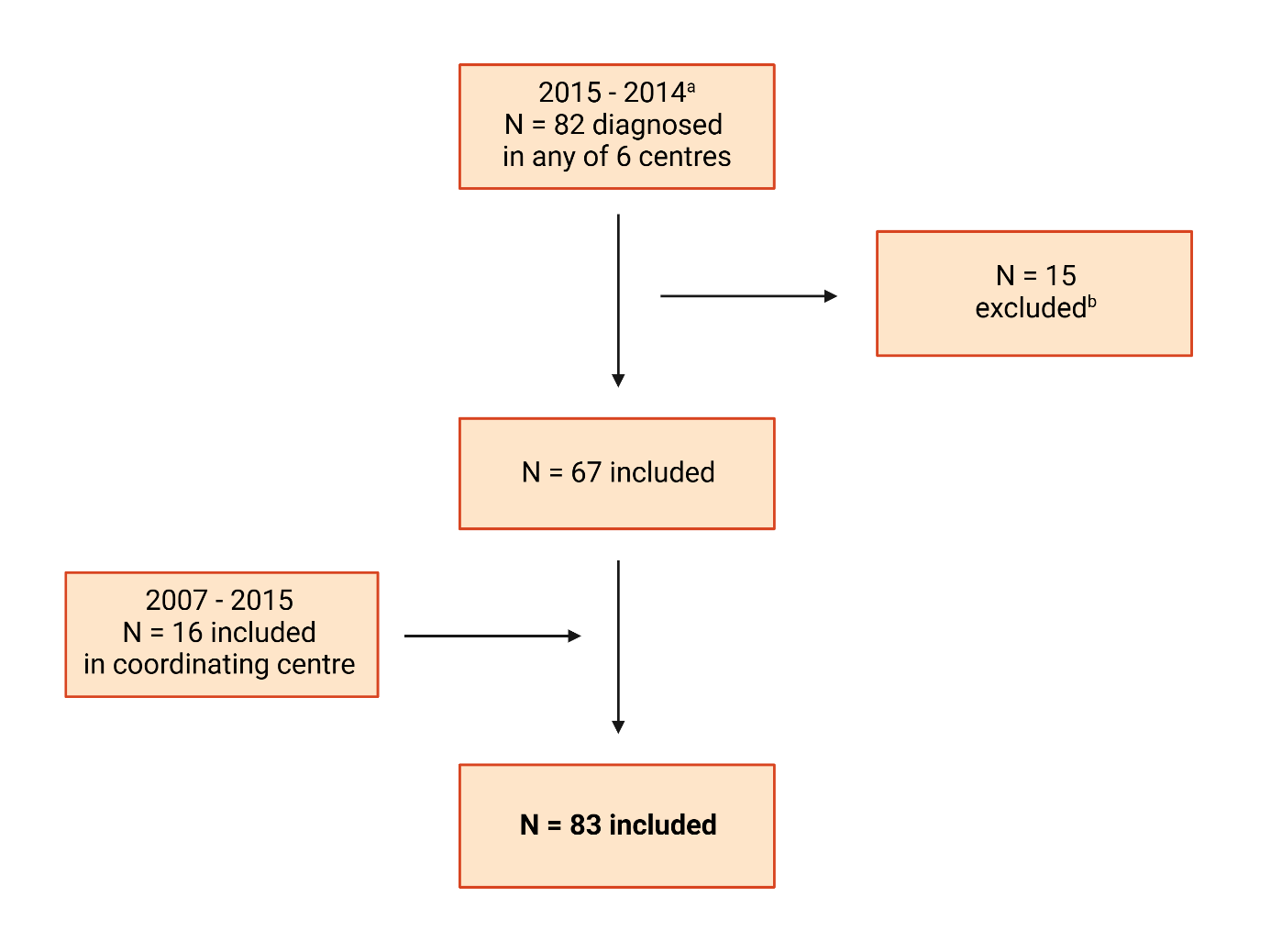


**Supplementary Figure S1. Flow diagram of study inclusions.** ^a^ Exact study period from June 2015 until October 2023. ^b^ 15 patients were not included because they were not approached for participation or declined to participate. Figure was created in BioRender.


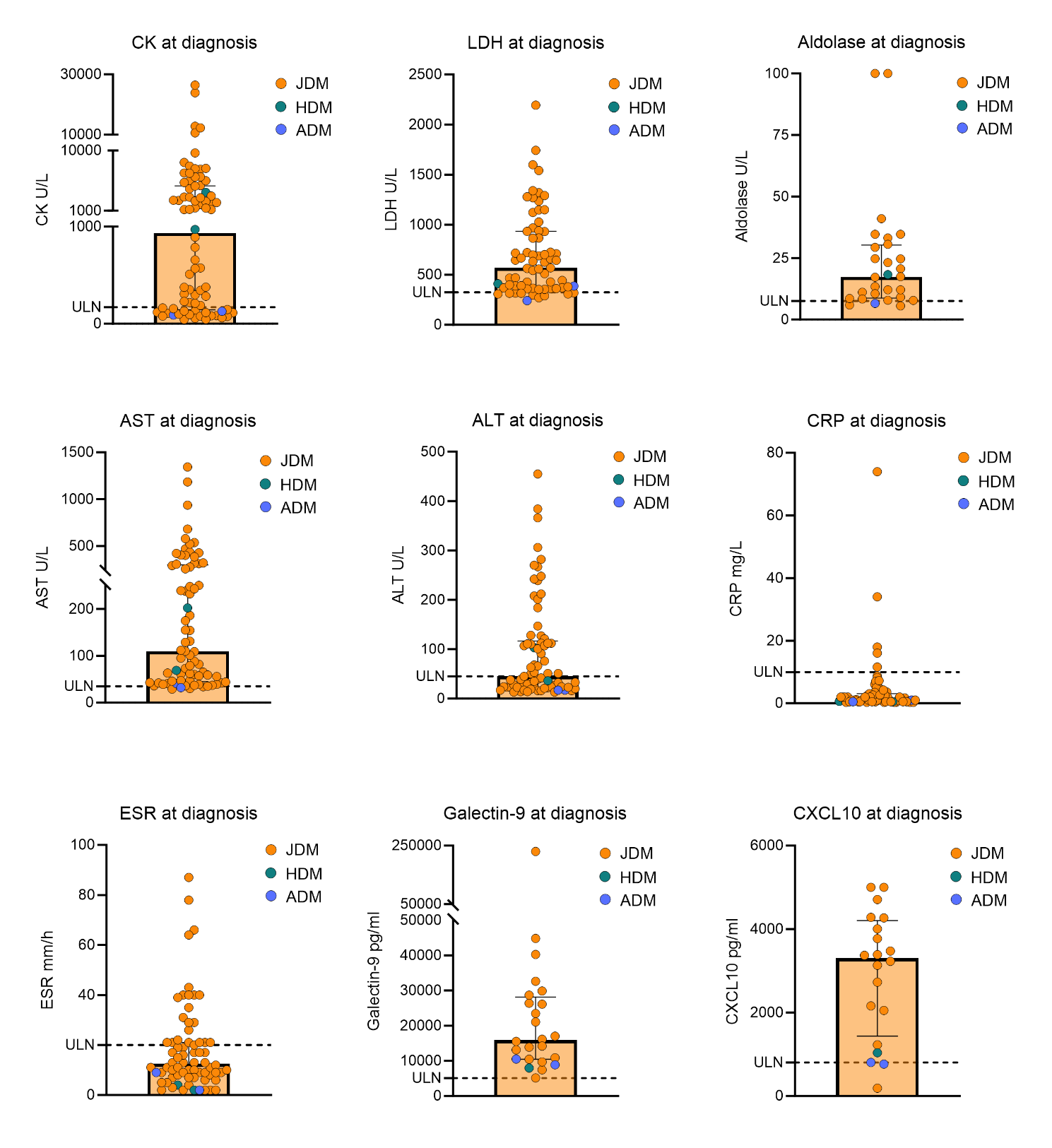


**Supplementary Figure S2. Blood test results at diagnosis.** Only Galectin-9 and CXCL10 levels measured in routine diagnostics are shown. HDM = hypomyopathic dermatomyositis, ADM = amyopathic dermatomyositis, ULN = upper limit of normal, CK = creatine kinase, LDH = lactate dehydrogenase, AST = aspartate transaminase, ALT = alanine transaminase.

**
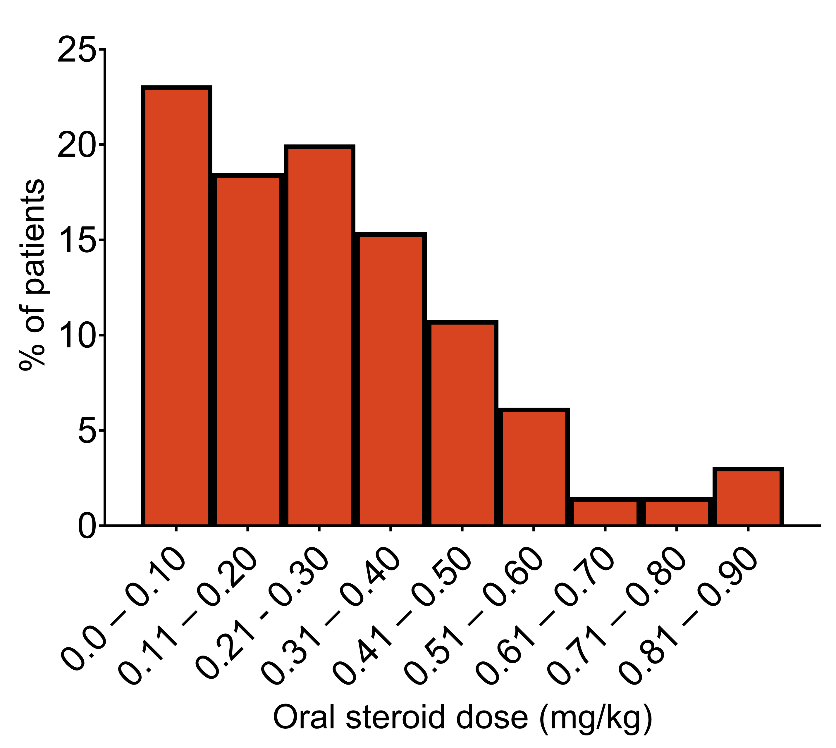
**

**Supplementary Figure S3. Oral steroid dose at 6-month follow-up.** Percentages of patients (n=65) receiving a certain oral steroid dose (mg/kg) at 6-month follow-up.


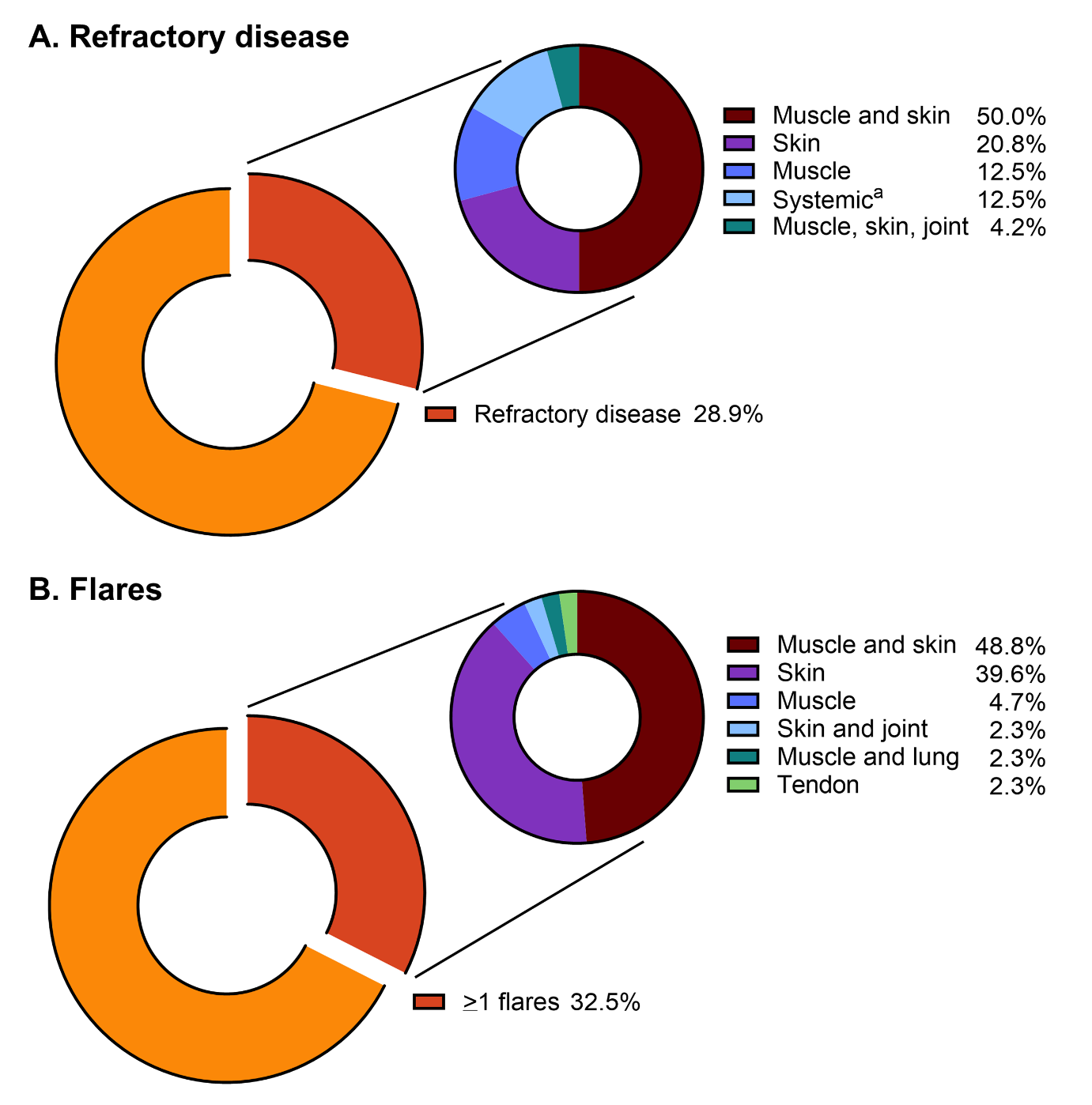


**Supplementary Figure S4. Refractory disease and flares.** Pie charts on the left show percentage of patients with refractory disease **(A)** or one or more flares **(B)** (n=83), while the pie charts on the right show which tissues were involved. ^a^ Systemic manifestations included generalized oedema, electrolyte imbalances, and thrombotic microangiopathy (n=1), fever (n=1) and generalized oedema and capillary leak (n=1).

**References**

1. Huber AM, Feldman BM, Rennebohm RM, Hicks JE, Lindsley CB, Perez MD, et al. Validation and clinical significance of the Childhood Myositis Assessment Scale for assessment of muscle function in the juvenile idiopathic inflammatory myopathies. Arthritis & Rheumatism. 2004;50(5):1595-603.

2. Huber AM, Lachenbruch PA, Dugan EM, Miller FW, Rider LG, For the Juvenile Dermatomyositis Disease Activity Collaborative Study G. Alternative scoring of the cutaneous assessment tool in juvenile dermatomyositis: Results using abbreviated formats. Arthritis Care & Research. 2008;59(3):352-6.

3. Wienke J, Bellutti Enders F, Lim J, Mertens JS, van den Hoogen LL, Wijngaarde CA, et al. Galectin-9 and CXCL10 as Biomarkers for Disease Activity in Juvenile Dermatomyositis: A Longitudinal Cohort Study and Multicohort Validation. Arthritis & Rheumatology. 2019;71(8):1377-90.
